# Data analyses on temperature-dependent behaviour of water based drilling fluid rheological models

**DOI:** 10.1016/j.dib.2018.09.100

**Published:** 2018-10-03

**Authors:** P.A.L. Anawe, J. Adewale Folayan

**Affiliations:** a Department of Petroleum Engineering, College of Engineering, Covenant University, Ota, Nigeria; b Department of Petroleum Engineering, University of Ibadan, Nigeria

**Keywords:** Ambient condition, Rheological models, OFITE rotational viscometer, Chandler HTHP viscometer, Water based mud

## Abstract

For this data article, the accuracy of different rheological models in estimation of rheological parameters of a bentonite-gel, water-based mud under ambient and elevated conditions were examined. The ambient conditions are pressure of 14.7 psia and 80 °F temperature while the elevated conditions are constant pressure of 5000 psi and different down hole temperatures of 120 °F, 160 °F and 200 °F. An OFITE, eight (8) speed rotational viscometer model 800 was used for ambient rheological properties measurement while a Chandler Model 7600 HTHP Viscometer was used to carry out rheological measurements at high temperature and pressure condition. The various rheological models under investigation are the Newtonian, Bingham Plastic, Power Law, American Petroleum Institute model (API 13D), Herschel–Bulkley, Unified and Casson Models.

## Specifications table

**Table:** 

Subject area	Chemical Engineering
More specific subject area	Fluid flow and transfer processes
Type of data	Tables, figures, images
How data was acquired	Experimental. The rheological properties of the mud sample were determined at temperature of 80 °F by using an OFITE, 8-speed rotational viscometer model 800 while a Chandler Model 7600 HTHP Viscometer was used to measure mud sample rheological properties at elevated temperatures of 120 °F, 160 °F and 200 F at constant pressure of 5000 psi. The viscometer has a rotational speed varying from 0 to 900 rpm with upper limit of pressure and temperature of 40,000 psig and 600 °F respectively. It also has an automatic 10-s and 10- min gel strength measurement.
Data format	Raw, Analyzed
Experimental factors	The rheology of fluids is strongly dependent on a number of factors such as temperature, pressure and prevailing shear rate. The various mathematical model used in the drilling fluid rheological characterization are Newtonian, Bingham Plastic, Power Law, API 13 D, Herschel-Bulkley, Unified and Casson Models.
Experimental features	A bentonite-gel water based mud was prepared in the laboratory under standard recommended procedures of American petroleum institute (API). The mud consists of fresh water as base fluid and bentonite as viscousity enhancing agent. Other additives include carboxyl methyl cellulose (CMC) as fluid loss control agent and potassium chloride (KCl) for inhibition of shale swelling and dispersion. Soda ash was added for treating possible calcium contamination while sodium hydroxide (NaOH) was used as PH control agent. Barite was also added as weighting agent.
Data source location	Drilling Fluid Research Laboratory, Portharcourt, Nigeria.
	Coordinates: 4°49′27″N, 7°2′1″E
Data accessibility	Data are available within this article
Related research article	None

## Value of the data

• The data are of immense benefit to the scientific community and researchers because it showed the effect of high temperature and pressure on drilling fluid rheological properties such as plastic viscosity, yield point and gel strength of water based drilling mud.
• The data provide useful information to the community of scientists and engineers by describing the flow behaviour (shear-thinning) phenomenon of water based drilling mud at high temperatures and pressures.
• The data will provide useful technical information to the scientific community and the petroleum industries on the best rheological model that can suitably represent water based mud. This information is very crucial in pressure losses and equivalent circulating density estimations.
• The data provides useful information to the petroleum industries on the accuracy of each rheological model stress values at high shear rate condition in the drill pipe and low shear rate condition in the bit.

## 1 Data

The data obtained from this research work comes from the experimental investigation of the effect of temperature on the rheological behaviour of water based drilling fluid. The data described the predictive ability of various drilling fluid rheological models in estimation of shear stress values at different shear rate conditions. Similarly, data on behaviour of each model at various temperatures and pressure conditions were also provided. Table 1 shows the rheological properties (shear stress, plastic viscosity, yield point and gel strength) of the formulated bentonite gel-water based mud at different temperatures while Table 2 describes the Newtonian rheological parameters at different temperatures. The Bingham Plastic yield stress ($\tau_{y})$ and plastic viscosity $(\mu_{p})$ are shown in Table 3 while Table 4 shows the Power Law flow behaviour index (*n*) and consistency index (*K*). The API model rheological parameters are shown in Table 5. Rheological parameters of Herschel–Bulkley and Unified rheological models are shown in Tables 6 and 7 respectively. The casson yield stress, plastic viscosity and casson stress model equations are described by Table 8.

**Table 1:** Rheological properties of water based mud in [lb/100 ft^2^] at different temperatures.

**Dial speed (RPM)**	**80 °F**	**120 °F**	**160 °F**	**200 °F**
**600**	82	61.5	44	32
**300**	55	42.5	31	23
**200**	43	33	24	19
**100**	34	25	18	15
**60**	25	19	13.5	10.5
**30**	20	14.5	10	7
**6**	15	10	8	5
**3**	11	7	5	3.5
**PV**	27	19	13	9
**YP**	28	23.5	18	14
**GEL (10 s)**	10	6	4	3
**GEL (10 min)**	13	8.5	6	4.5

**Table 2:** Newtonian model rheological parameters at various temperatures.

**Parameter**	**80 °F**	**120 °F**	**160 °F**	**200 °F**
μ (lb/100 ft^2^ s)	0.093	0.0703	0.0506	0.0376
τ**(lb/100 ft**^**2**^**)**	**0.093**γ	**0.0703**γ	**0.0506**γ	**0.0376**γ

**Table 3:** Bingham plastic model rheological parameters at various temperatures.

**Parameter**	**80 °F**	**120 °F**	**160 °F**	**200 °F**
***τ_y_* (Pa)**	14.308	12.008	9.198	7.154
***µ_p_* (Pa s)**	0.0138	0.0097	0.00664	0.004599
***τ* (Pa)**	14.308+0.0138γ	12.008+0.0097γ	9.198+0.00664γ	7.154+0.004599γ

**Table 4:** Power law model rheological parameters at various temperatures.

**Parameter**	**80 °F**	**120 °F**	**160 °F**	**200 °F**
***n***	0.576	0.533	0.505	0.476
***K* (lb/100 ft**^**2**^**)**	1.5146	1.5304	1.329	1.182
***τ* (Pa)**	$\tau =0.774\gamma^{0.576}$	$\tau =0.782\gamma^{0.533}$	$\tau =0.679\gamma^{0.505}$	$\tau =0.604\gamma^{0.476}$

**Table 5:** API model rheological parameters at various temperatures.

**Parameter**	**80 °F**	**120 °F**	**160 °F**	**200 °F**
**Drill Pipe**				
$n_{\mathrm{pipe}}$	0.576	0.533	0.505	0.476
***k**_**pipe**_***(lb/100 ft**^**2**^**)**	1.5146	1.5304	1.329	1.182
$\tau_{\mathrm{pipe}}$**(Pa)**	$0.774\gamma^{0.576}$	$0.782\gamma^{0.533}$	$0.679\gamma^{0.505}$	$0.604\gamma^{0.476}$
**Annulus**				
$n_{\mathrm{annulus}}$	**0.3220**	**0.3632**	**0.3655**	**0.4152**
***K**_**a**_***(lb/100 ft**^**2**^**)**	**6.5017**	**3.8687**	**2.7527**	**1.7770**
$\tau_{\mathrm{annulus}}$**(Pa)**	$3.3224\gamma^{0.322}$	$1.9769\gamma^{0.3632}$	$1.4066\gamma^{0.3655}$	$0.9081\gamma^{0.4152}$

**Table 6:** Herschel–Bulkley model rheological parameters at various temperatures.

**Parameter**	**80 °F**	**120 °F**	**160 °F**	**200 °F**
***τ_oH_* (Pa)**	5.621	3.577	2.555	1.788
$n_{H}$	0.6335	0.6366	0.5831	0.664
***k**_**H**_***(pa)(Pa s)**	0.4236	0.3340	0.3263	0.1595
***τ* (Pa)**	$5.621+0.4236\gamma^{0.6335}$	$3.577+0.3340\gamma^{0.6366}$	$2.555+0.3263\gamma^{0.5831}$	$1.788+0.1595\gamma^{0.664}$

**Table 7:** Unified model rheological parameters at various temperatures.

**Parameter**	**80 °F**	**120 °F**	**160** **°F**	**200 °F**
**Drill Pipe**				
$\tau_{o}$**(lb/100 ft**^**2**^)	7.462	4.264	2.132	2.132
$n_{\mathrm{pipe}}$	0.576	0.5331	0.505	0.476
***k**_**p**_***(lb/100 ft**^**2**^**)**	1.6146	1.6304	1.4170	1.260
$\tau_{\mathrm{pipe}}$**(Pa)**	$3.813+0.8251\gamma^{0.576}$	$2.179+0.8331\gamma^{0.5331}$	$1.089+0.7241\gamma^{0.505}$	$1.089+0.6439\gamma^{0.476}$
**Annulus**				
$n_{\mathrm{annulus}}$	0.6489	0.5820	0.5364	0.5173
***K**_**a**_***(lb/100 ft**^**2**^**s**^**n**^**)**	0.8857	1.0813	1.0849	0.8834
$\tau_{\mathrm{annulus}}$**(Pa)**	$3.813+0.4526\gamma^{0.6489}$	$2.179+0.5525\gamma^{0.5820}$	$1.089+0.5543\gamma^{0.5364}$	$1.089+0.4514\gamma^{0.5173}$

**Table 8:** Casson model rheological parameters at various temperatures.

**Parameter**	**80 °F**	**120 °F**	**160 °F**	**200 °F**
***K**_**oc**_***(Pa)**	5.0312	3.3528	2.4712	1.7563
***K**_**c**_***(MPa s)**	0.0180	0.01492	0.01058	0.008278
***τ* (Pa)**	${(2.243+0.1342\gamma^{0.5})}^{2}$	${(1.831+0.1221\gamma^{0.5})}^{2}$	${(1.572+0.1029\gamma^{0.5})}^{2}$	${(1.325+0.0910\gamma^{0.5})}^{2}$

**Fig. 1:**
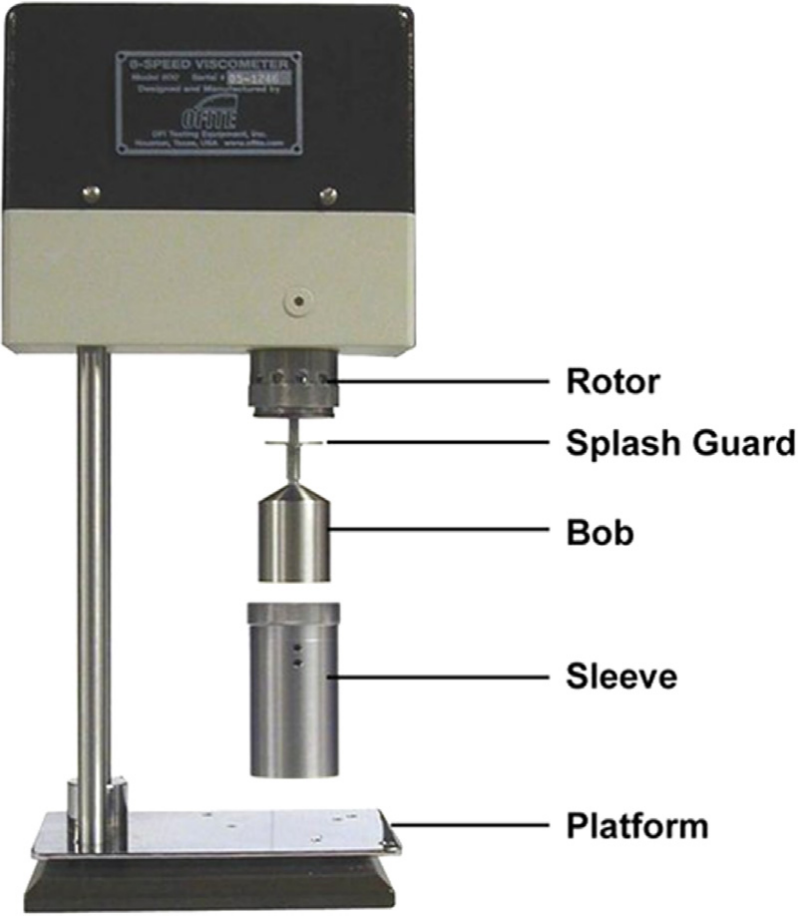
OFITE-8 speed rotational viscometer model 800.

**Fig. 2:**
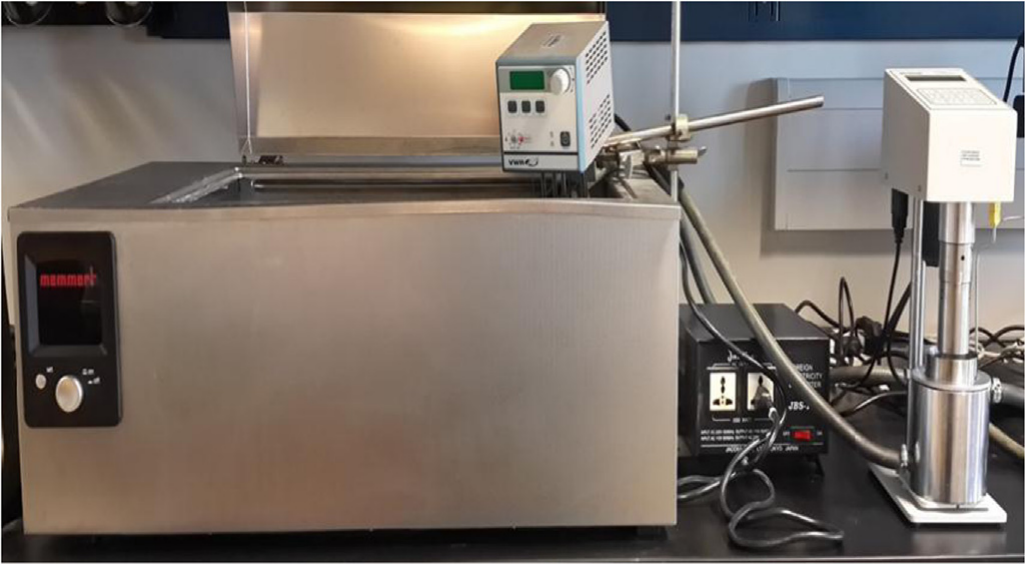
Chandler (Model 7600) HPHT viscometer.

**Fig. 3:**
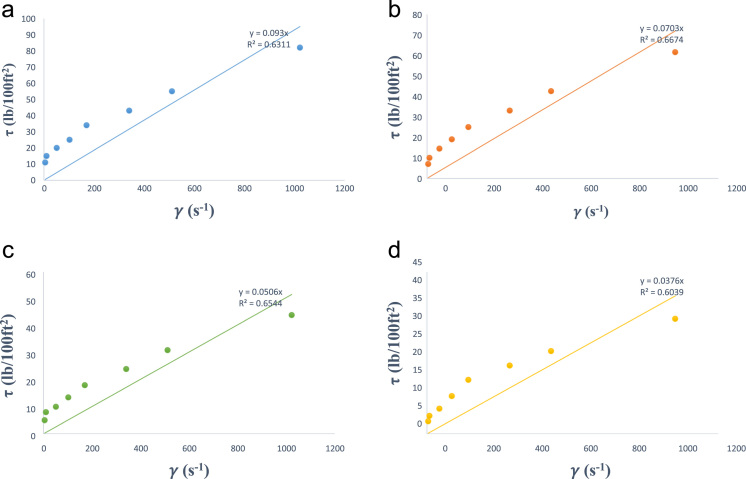
a: Newtonian rheological model plot at 80 °F. b: Newtonian rheological model plot at 120 °F. c: Newtonian rheological model plot at 160 °F. d: Newtonian rheological model plot at 200 °F.

Fig. 1 is the pictorial representation of an OFITE, 8-speed rotational viscometer model 800 for stress values measurement at ambient conditions while Fig. 2 represents Chandler (Model 7600) HPHT Viscometer for high-temperature, high-pressure shear stress measurement. The Newtonian rheological plot for the estimation of fluid viscosity at various temperatures is shown in Fig. 3a–d. Fig. 5a–d represents the logarithmic plot of **(*τ*–*τ****_**oH**_***)** versus **(*γ*)** at different temperatures for the estimation of Herschel–Bulkley flow behaviour index **(*n****_**H**_***)** and consistency factor **(*k****_**H**_***)**. Finally, Fig. 5a–d is the plot of square root of shear stress **(*τ***^**0.5**^**)** against the square root of shear rate **(*γ***^**0.5**^**)** for the deduction of casson yield stress **(*k****_**oc**_***)** and plastic viscosity **(*k****_**c**_***)**.

**Fig. 4:**
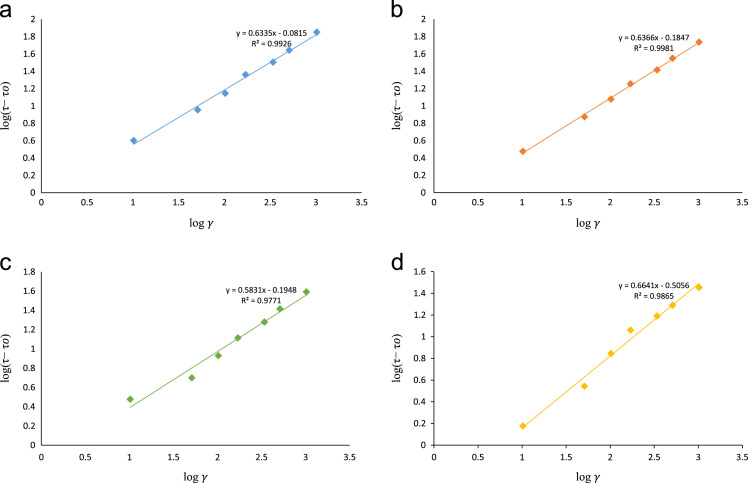
a: Herschel–Bulkley rheological model plot at 80 °F. b: Herschel–Bulkley rheological model plot at 120 °F. c: Herschel–Bulkley rheological model plot at 160 °F. d: Herschel–Bulkley rheological model plot at 200 °F.

**Fig. 5:**
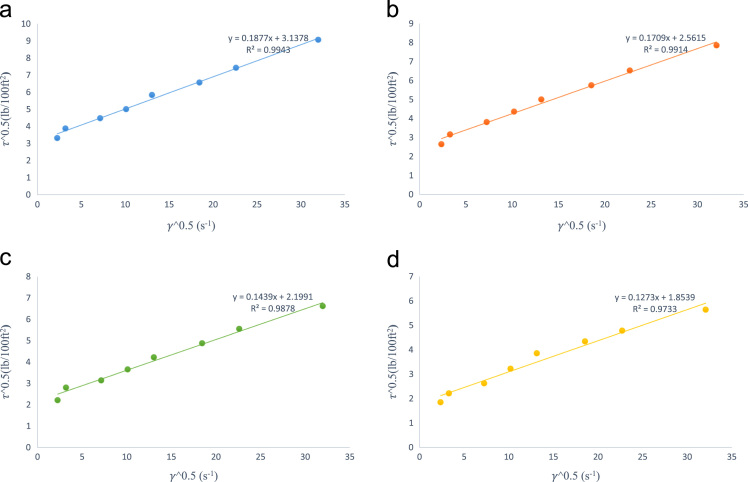
a: Casson rheological model plot at 80 °F. b: Casson rheological model plot at 120 °F. c: Casson rheological model plot at 160 °F. d: Casson rheological model plot at 200 °F.

**Fig. 6:**
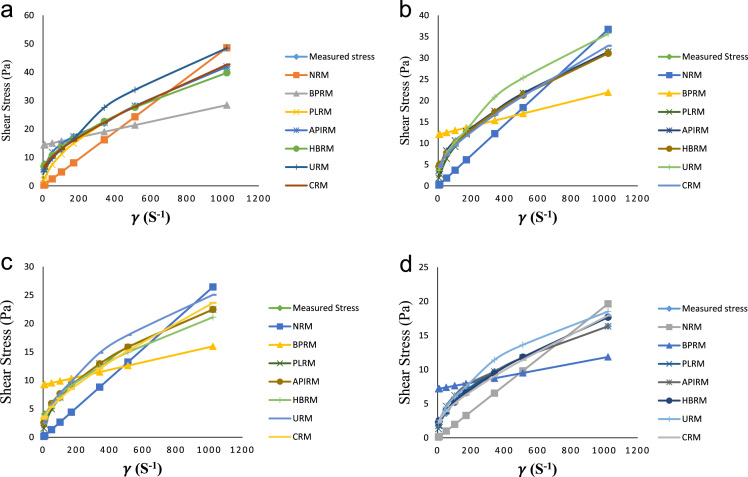
a: Plot of measured stress and predicted stress by different rheological models at 80 °F. b: Plot of measured stress and predicted stress by different rheological models at 120 °F. c: Plot of measured stress and predicted stress by different rheological models at 160 °F. d: Plot of measured stress and predicted stress by different rheological models at 200 °F.

The plot of measured stress and predicted stress by different rheological models at 80 °F, 120 °F, 160 °F and 200 °F are shown by Fig. 6a–d respectively.

## 2 Experimental design, materials and methods

### 2.1 Mud sample preparation and rheology measurement

A bentonite-gel water based mud was prepared in the laboratory under standard recommended procedures of American petroleum institute (API). The mud consists of fresh water as base fluid and bentonite as viscousity enhancing agent. Other additives include carboxyl methyl cellulose (CMC) as fluid loss control agent and potassium chloride (KCl) for inhibition of shale swelling and dispersion. Similarly, Soda ash was added for treating possible calcium contamination while sodium hydroxide (NaOH) was used as PH control agent. Barite was added as weighting agent with some biopolymer as rheology modifier. The rheological properties of the mud sample were determined at temperature of 80°F by using an OFITE, 8-speed rotational viscometer model 800 shown in Fig. 1 while Chandler Model 7600 HTHP Viscometer shown in Fig. 2 was used to measure mud sample rheological properties at elevated temperatures of 120 °F, 160 °F and 200 °F at constant pressure of 5000 psi. This Viscometer is designed for determining the rheology of drilling fluids while subjected to varying well conditions in accordance with International Standard Organization (ISO) and American Petroleum Institute (API) standards. The viscometer has a rotational speed varying from 0 to 900 rpm with upper limit of pressure and temperature of 40,000 psig and 600 °F respectively. It also has an automatic ten-seconds and ten-minutes gel strength measurement.

### 2.2 Drilling fluid rheological models

#### 2.2.1 Newtonian rheological model

The Newtonian model [1] assumes that shear stress (*τ*) is directly proportional to the shear rate (*γ*) and the constant of proportionality is the fluid viscosity (*µ*). The shear stress can be mathematically described by Eq. (1).

(1) τ=µ*γ

#### 2.2.2 Bingham plastic rheological model

The Bingham plastic model [2] is a two parameter model that is mathematically represented by Eq. (2). Bingham Plastic fluids are characterized by a yield stress (*τ_y_*) and plastic viscosity (*µ_p_*) that are not shear rate dependent [3].

(2) $$\tau =\tau_{y}+\mu_{p}\gamma$$

Where $\tau_{y}$ is called the yield stress and the unit is lb/100 ft^2^ or Pa, $\mu_{p}$ is the plastic viscosity of the fluid in mPaS (cp), and γ is the shear rate (s^-1^).

(3) $$\tau_{y}=\theta_{300}-{µ}_{p}$$

(4) $${µ}_{p}=\theta_{600}-\theta_{300}$$

#### 2.2.3 Power law rheological model

The power law model [4] is expressed by Eq. (5)

(5) $$\tau ={k\gamma }^{n}$$

where *n* is the dimensionless flow behaviour index which is an indicator of the shear thinning or thickening nature of the fluid [5] and *k* is the consistency factor in lb/100 ft^2^ which can be converted to pascal through multiplying by 0.511 conversion factor [6].

(6) $$n=3.322\;\log\left(\frac{\theta_{600}}{\theta_{300}}\right)$$

(7) $$k=\frac{\theta_{300}}{511^{n}}$$

#### 2.2.4 API rheological model

The API rheological model was proposed as a panacea to the underestimation error that is associated with power law rheological model at low shear rate condition in the annulus. Hence a new equation was modelled to describe drilling fluid shear stress in the annulus [7].

(a) **Drill Pipe**
  (8) $$\tau_{\mathrm{pipe}}={k_{p}\gamma }^{n_{p}}$$
  (9) $$n_{p}=3.322\;\log\left(\frac{\theta_{600}}{\theta_{300}}\right)$$
  (10) $$k_{p}=\frac{\theta_{300}}{511^{n_{p}}}$$
(b) **Annulus**
  (11) $$\tau_{\mathrm{annulus}}={k_{a}\gamma }^{n_{a}}$$
  (12) $$n_{a}=0.657\;\log\left(\frac{\theta_{100}}{\theta_{3}}\right)$$
  (13) $$k_{a}=\frac{\theta_{100}}{170.3^{n_{a}}}$$

#### 2.2.5 Herschel–Bulkley rheological model

The Herschel–Bulkley Model is an extension of the Bingham Plastic model to include shear rate dependence [8]. The model as proposed by [9] is given by Eq. (14).

(14) $$\tau =\tau_{\mathrm{OH}}+k_{H}\gamma^{n_{H}}$$

Where *γ* is the shear rate (s^-1^), *τ* is the shear stress (Pa), *n_H_* is the flow behaviour index (dimensionless) and *k_H_* is the HRBM consistency index in (Pa) and *τ_OH_* is the HBRM yield stress (Pa).

A plot of log (*τ*–*τ_oH_*) versus log (*γ*) as shown in Fig. 4a–d will result in a straight line with intercept log *k_H_* and slope *n_H_* respectively.

#### 2.2.6 Unified rheological model

The unified model equation is a more correct form of the Herschel–Bulkley rheological model. The mathematical form is represented by Eqs. (15) and (19) for drill pipe and annulus respectively [10].

(a) **Drill Pipe**
  (15) $$\tau_{\mathrm{pipe}}={{\tau_{O}+k}_{p}\gamma }^{n_{p}}$$
  (16) $$\tau_{O}=1.066(2\theta_{3}-\theta_{6})$$
  (17) $$n_{p}=3.322\;\log\left[\frac{2{µ}_{p}+\tau_{y}}{{µ}_{p}+\tau_{y}}\right]$$
  (18) $$k_{p}=1.066\left[\frac{{µ}_{p}+\tau_{y}}{511^{n_{p}}}\right]$$
(b) **Annulus**
  (19) $$\tau_{\mathrm{annulus}}={\tau_{O}+k_{a}\gamma }^{n_{a}}$$
  (20) $$n_{a}=3.322\;\log\left[\frac{2{µ}_{p}+\tau_{y}-\tau_{O}}{{µ}_{p}+\tau_{y}-\tau_{O}}\right]$$
  (21) $$k_{a}=1.066\left[\frac{{µ}_{p}+\tau_{y}-\tau_{O}}{511^{n_{a}}}\right]$$

#### 2.2.7 Casson Rheological Model

The Casson Rheological Model [11] is a structure based model that is used to describe the flow of visco-elastic fluids. This model has a more gradual transition from Newtonian to the Yield region. Mathematically, the Casson model is expressed as

(22) $$\tau^{\frac{1}{2}}=k_{\mathrm{oc}}^{\frac{1}{2}}+k_{c}^{\frac{1}{2}}\gamma^{\frac{1}{2}}$$

where *k_oc_* is Casson yield stress (Pa), *k_c_* is Casson plastic viscosity in Pa s.

The parameters *k_oc_* and *k_c_* can be obtained from the straight line that is drawn when the square root of shear stress (*τ*^0.5^) is plotted against the square root of shear rate (*γ*^0.5^) [Fig. 5a–d] with the slope *k_c_* and intercept *k_oc_*.
